# Self-harm-related mental health presentations to emergency departments by children and young people from culturally and linguistically diverse groups in South Western Sydney

**DOI:** 10.1192/bjo.2024.763

**Published:** 2024-12-04

**Authors:** James Rufus John, Jahidur Rahman Khan, Paul M. Middleton, Yao Huang, Daniel Ping-I Lin, Nan Hu, Bin Jalaludin, Paul Chay, Raghu Lingam, Valsamma Eapen

**Affiliations:** School of Clinical Medicine, University of New South Wales, Sydney, New South Wales, Australia; and Ingham Institute of Applied Medical Research, Liverpool, New South Wales, Australia; School of Clinical Medicine, University of New South Wales, Sydney, New South Wales, Australia; Ingham Institute of Applied Medical Research, Liverpool, New South Wales, Australia; South Western Emergency Research Institute, Liverpool, New South Wales, Australia; and School of Population Health, University of New South Wales, Sydney, New South Wales, Australia; Ingham Institute of Applied Medical Research, Liverpool, New South Wales, Australia; and South Western Emergency Research Institute, Liverpool, New South Wales, Australia; School of Clinical Medicine, University of New South Wales, Sydney, New South Wales, Australia; and South Western Sydney Local Health District, Liverpool, New South Wales, Australia; Ingham Institute of Applied Medical Research, Liverpool, New South Wales, Australia; and School of Population Health, University of New South Wales, Sydney, New South Wales, Australia; School of Clinical Medicine, University of New South Wales, Sydney, New South Wales, Australia; Ingham Institute of Applied Medical Research, Liverpool, New South Wales, Australia; and South Western Sydney Local Health District, Liverpool, New South Wales, Australia

**Keywords:** Self-harm, emergency department, adolescents, culturally and linguistically diverse (CALD) background, mental health

## Abstract

**Background:**

Rates of self-harm among children and young people (CYP) have been on the rise, presenting major public health concerns in Australia and worldwide. However, there is a scarcity of evidence relating to self-harm among CYP from culturally and linguistically diverse (CALD) backgrounds.

**Aims:**

To analyse the relationship between self-harm-related mental health presentations of CYP to emergency departments and CALD status in South Western Sydney (SWS), Australia.

**Method:**

We analysed electronic medical records of mental health-related emergency department presentations by CYP aged between 10 and up to 18 years in six public hospitals in the SWS region from January 2016 to March 2022. A multilevel logistic regression model was used on these data to assess the association between self-harm-related presentations and CALD status while adjusting for covariates and individual-level clustering.

**Results:**

Self-harm accounted for 2457 (31.5%) of the 7789 mental health-related emergency department presentations by CYP; CYP from a CALD background accounted for only 8% (*n* = 198) of the self-harm-related presentations. CYP from the lowest two most socioeconomic disadvantaged areas made 63% (*n* = 1544) of the total self-harm-related presentations. Findings of the regression models showed that CYP from a CALD background (compared with those from non-CALD backgrounds) had 19% lower odds of self-harm (adjusted odds ratio 0.81, 95% CI 0.66–0.99).

**Conclusions:**

Findings of this study provide insights into the self-harm-related mental health presentations and other critical clinical features related to CYP from CALD backgrounds that could better inform health service planning and policy to manage self-harm presentations and mental health problems among CYP.

Self-harm among children and young people (CYP) is a major public health concern in Australia and worldwide, with far-reaching consequences for individuals, families and communities both in the short term and over time.^1^ This is particularly concerning as the rates of self-harm among Australian young people have increased by 35% between 2010 and 2021.^2^ Further, an Australian survey reported that around 11% of CYP (aged 12–17) have engaged in self-harm at some point in their lives.^2^ There is also substantial evidence suggesting that a history of self-harm increases the risk of repeat self-harm or suicide.^3,4^ In Australia, suicide is the leading cause of death in CYP, with a national rate of 2.3 per 100 000 among those aged 5–17 years.^5^ CYP are particularly vulnerable and experience a disproportionate impact from the effects of self-harm and suicide-related behaviours, often due to adverse early childhood experiences, social media, bullying at school, family conflicts, etc.^6^ Studies show that self-harm may lead to adverse emotional and physical outcomes resulting in episodes of major depression and other mental health problems^7^ as well as serious physical injuries and poor quality of life^8^ that can have long-lasting effects on their overall health and social well-being in adulthood.^9^ Besides health ramifications, self-harm can incur a significant economic burden, with recent evidence estimating the total economic loss attributable to youth suicide in Australia at $22 billion a year.^10^

It is worth noting that CYP from culturally and linguistically diverse (CALD) backgrounds (also referred to as those from a non-English-speaking background) are disproportionately affected by self-harm and suicidal behaviour. CALD status acknowledges individuals of various ethnicities, languages and cultural practices within a broader societal framework. In accordance with existing literature and nationally consistent indicators,^11^ CALD status has been defined using three approaches: (a) if the person's preferred language is non-English, (b) if the person's country of birth is a non-English-speaking country and (c) if the person's preferred language is non-English and/or the country of birth is a non-English-speaking country (composite CALD status). CYP from CALD backgrounds face an elevated risk of self-harm and suicidal behaviour which has been attributed to specific factors such as cultural stigmas, taboos and language barriers, which may impede their ability to seek help and hinder effective public health communication and relevant supports.^12^ For instance, a study conducted in Western Australia found that 61% of adolescent refugees with a background in resettlement exhibited health concerns related to suicide and mental health issues. An examination of self-harm-related hospital admissions among individuals aged 15 and older in Victoria, Australia, between 2014 and 2019 revealed that rates were significantly lower among those from CALD background but that there were substantial variations depending on mode of entry into Australia, such as refugee versus planned migration.^13^ However, this study did not include emergency department presentation data or regions with a high density of multicultural communities. Moreover, only a few international studies^14^ have focused solely on both CALD populations and adolescents aged 10–17, a crucial period characterised by substantial physical, emotional, intellectual and social transformations, thereby lacking local context. Hence, further research is needed to delineate the disparities in self-harm-related mental health emergency department presentations by CYP from CALD population groups alongside key social determinants of health – a key objective of this study.

Emergency departments serve as the initial point of contact for CYP who engage in self-harm.^15^ Despite the presence of various community-based mental health services, there is evidence indicating an increasing number of CYP seeking emergency department care for self-harm and other mental health problems.^16,17^ Previous studies have reported that adolescents with self-harm-related emergency department presentations were more likely to attempt, and to die by, suicide compared with those who do not present to the emergency department.^18^ Presentations to the emergency department thus offer potentially life-saving opportunities for intervention and prevention. However, there is a scarcity of literature on self-harm-related emergency department presentations by CYP from CALD backgrounds in Australia.

To address these knowledge gaps, we aimed to investigate the self-harm-related emergency department presentations of CYP from CALD backgrounds in six public hospitals in the South Western Sydney Local Health District (SWSLHD) over a period from 2016 to 2022. SWSLHD is home to a multicultural population, with 43% of the South Western Sydney (SWS) residents born overseas, compared with the New South Wales (NSW) state average of 34%,^19^ and a much higher percentage of CYP (31.3% compared with the state average of 24.5%). Furthermore, social disadvantage (e.g. lower levels of educational attainment and income) is also notably high in this region compared with other parts of NSW, with a national report^20^ featuring three suburbs in SWS in the list of top 10 communities most severely affected by COVID-19 and experiencing family employment stress. In keeping with the findings from previous studies, we hypothesised that there will be a significant association between self-harm-related mental health emergency department presentations and CALD status. Understanding the direction and strength of this association in the context of key social determinants of health is expected to inform health service planning and policy to better manage self-harm presentations and mental health problems among CYP from CALD backgrounds.

## Method

### Study design and data source

We analysed prospectively collected de-identified electronic medical records (eMRs) of paediatric mental health-related emergency department presentations from six public hospitals in SWSLHD between January 2016 and March 2022. The STROBE checklist was used as the guideline for this study.

### Cohort selection

This study comprised encounter-level information on CYP aged 10 to 17 years who presented to the emergency department for a mental health problem in one of the six SWSLHD public hospitals during the study period. Secure access to the eMR data was enabled via the Comprehensive Emergency Dataset for Research, Innovation and Collaboration (CEDRIC) platform, which draws data from the eMR system captured during service interactions with opportunities to track service use. The data sourced from the CEDRIC platform comprised demographic data, dates/times of care, triage classification and discharge diagnosis coded in the Systematised Nomenclature of Medicine–Clinical Terminology Australian Extension (SNOMED CT-AU). The snoMAP-Starter AU tool was used to convert the SNOMED CT-AU codes into the corresponding ICD-10 Australian Modification (ICD-10-AM) codes.^21^ If an ICD-10-AM code for a mental health condition was present for an encounter, we classified this encounter as having a mental health-related presentation.^21^ A list of ICD-10-AM codes used to classify encounters with mental disorders in this database was reported in earlier research.^22^ When extracting data from CEDRIC, we excluded duplicate presentations (same encounter date and time of presentations) resulting from ambiguous encounter information recorded in the eMR. Furthermore, because this study focused on emergency department presentations rather than individuals, the same person could have had multiple encounters. We analysed 7789 mental health-emergency department presentations made by 4708 individual CYP during the study period.

### Outcome measure, exposure and covariates

Self-harm-related mental health emergency department presentation was the primary outcome variable of interest using the ICD-10-AM codes: X60–X84, Y10–Y34, Y87.0, Y87.2, Z91.5 and R45.81 (Supplementary Table 1, available at https://doi.org/10.1192/bjo.2024.763). A mental health-related presentation was marked as ‘yes’ if it was a self-harm-related condition and ‘no’ otherwise.

The primary exposure variables included CALD status adapted from the Australian Bureau of Statistics (ABS) definition of CALD using the minimum core set of cultural (defined by country of birth as English speaking/non-English speaking) and/or language indicators (defined by preferred language).

A set of variables were regarded as covariates in accordance with the literature and health inequity theory with a particular focus on social determinants of health.^23^ These variables were: age at presentation to the emergency department (in years), gender (male, female, undisclosed), day of arrival (weekday, weekend), residential area (i.e. postcode), socioeconomic status (least advantaged, second least advantaged, second most advantaged, most advantaged), area remoteness classification (major cities, inner or outer regions) and COVID-19 period (pre-COVID-19 from January 2016 to February 2020, during COVID-19 from March 2020 to March 2022).

The socioeconomic status of patients’ residential postcodes was calculated using the ABS Index of Relative Socioeconomic Disadvantage (IRSD) for the 2016 census population and divided into quartiles in this study. This study classified the remoteness of each patient's residential postcodes using the Accessibility and Remoteness Index of Australia (ARIA), which was created by the ABS to measure access to services. Areas were divided into five classes: major cities of Australia, inner regional Australia, outer regional Australia, remote and very remote. Since no patients’ postcodes were found in the remote or very remote categories, the postcodes used in this study were divided into three categories (major cities and inner and outer regional Australia).

### Statistical analysis

Descriptive statistics were initially used to compare the proportion of self-harm-related mental health emergency department presentations across key covariates and by CALD status. The primary analysis included a multilevel binary logistic regression model to evaluate the association between self-harm-related mental health presentation and the exposure variables/covariates, while controlling for individual-level clustering. Since a notable number of people had multiple mental health-related presentations, patient-level clustering was adjusted to estimate more accurate standard error of parameters. At first, we used unadjusted models to examine the one-to-one relationship between the exposure variable/covariate and the outcome. Next, we used adjusted models that considered the exposure variable and outcome variable, while also accounting for covariates. Multiple models were fitted to assess the impact of varying definitions of CALD on the associations between CALD status and outcome variable. Four models were developed: (a) CALD status (based on preferred language and/or country of birth) + covariates; (b) country of birth + covariates; (c) preferred language + covariates; and (d) preferred language + country of birth + covariates. The results are presented as crude odds ratios (COR) for unadjusted models and as adjusted odds ratios (AOR) for adjusted models from regression analysis, together with their respective 95% confidence intervals (95% CI). Furthermore, the variance inflation factor was used to evaluate multicollinearity when a single model included both the preferred language and the country of birth. R version 3.6.3 for Windows was used to conduct statistical analysis.

### Ethics approval

The authors assert that all procedures contributing to this work comply with the ethical standards of the relevant national and institutional committees on human experimentation and with the Helsinki Declaration of 1975, as revised in 2008. The study was approved by the Institutional Review Ethics Committee of SWSLHD Human Research Ethics Committee (2019_ETH12073). Given the use of electronic medical records, a waiver of consent was granted by the ethics committee.

## Results

### Overall mental health-related emergency department presentations

Descriptive characteristics of the overall mental health-related and self-harm-related emergency department presentations of CYP (age at mental health-related emergency department presentation 10–17 years) are presented in Table 1. For the overall mental health-related presentations, the average age was 14.9 years (s.d. = 1.8) and 63.2% of patients were female. In terms of the country of birth, only 6.9% of mental health-related presentations were made by CYP born in non-English-speaking countries, 4.6% were made by CYP whose preferred language was other than English, and 9.2% were made by CYP with CALD background. About 50.3% of mental health presentations were made by CYP residing in the most socioeconomically deprived areas (quartile 1) and 12.5% by CYP residing in inner or outer regional areas. When compared with the weekends, the weekdays had a higher proportion of mental health-related presentations (77.0%). Furthermore, 37.3% of all mental health-related presentations occurred during the COVID-19 period.

**Table 1:** Distribution of all mental health and self-harm related emergency department presentations by children and young people (10–17 years of age) by exposures and covariates

Characteristics	All mental health presentations (*n* = 7789) (column %)	Unique number of patients	Self-harm related mental health presentations (*n* = 2457; 31.5%) (row %)
Age at emergency department presentation, years: mean (s.d.)	14.9 (1.82)	4708	15.0 (1.66)
Gender			
Male	2812 (36.1%)	1860	771 (27.4%)
Female	4923 (63.2%)	2832	1659 (33.7%)
Undisclosed	54 (0.69%)	16	27 (50.0%)
CALD background			
Yes	717 (9.21%)	512	198 (27.6%)
No	7071 (90.8%)	4195	2258 (31.9%)
Unknown	1 (0.01%)	1	1 (100.0%)
Country of birth			
English-speaking country	7243 (93.0%)	4318	2306 (31.8%)
Non-English-speaking country	534 (6.9%)	379	148 (27.7%)
Unknown	12 (0.15%)	11	3 (25.0%)
Preferred language			
English	7417 (95.2%)	4450	2370 (32.0%)
Non-English	361 (4.6%)	248	85 (23.5%)
Unknown	11 (0.14%)	10	2 (18.2%)
Residential area socioeconomic status			
Q1: Least advantaged (poorest)	3921 (50.3%)	2351	1232 (31.4%)
Q2: 2nd least advantaged	944 (12.1%)	570	312 (33.1%)
Q3: 2nd most advantaged	1174 (15.1%)	723	358 (30.5%)
Q4: most advantaged (wealthiest)	1566 (20.1%)	959	486 (31.0%)
Unknown	184 (2.4%)	105	69 (37.5%)
COVID-19 period			
Pre-COVID	4881 (62.7%)	3110	1420 (29.1%)
During COVID	2908 (37.3%)	1885	1037 (35.7%)
Day of arrival			
Weekday	5996 (77.0%)	3808	2005 (33.4%)
Weekend	1793 (23.0%)	1458	452 (25.2%)
Residential area remoteness			
Major cities	6634 (85.2%)	4013	2092 (31.5%)
Inner or outer regions	971 (12.5%)	590	296 (30.5%)
Unknown	184 (2.4%)	105	69 (37.5%)

Q, quartiles; CALD, culturally and linguistically diverse.

### Self-harm-related mental health emergency department presentations

**Fig. 1:**
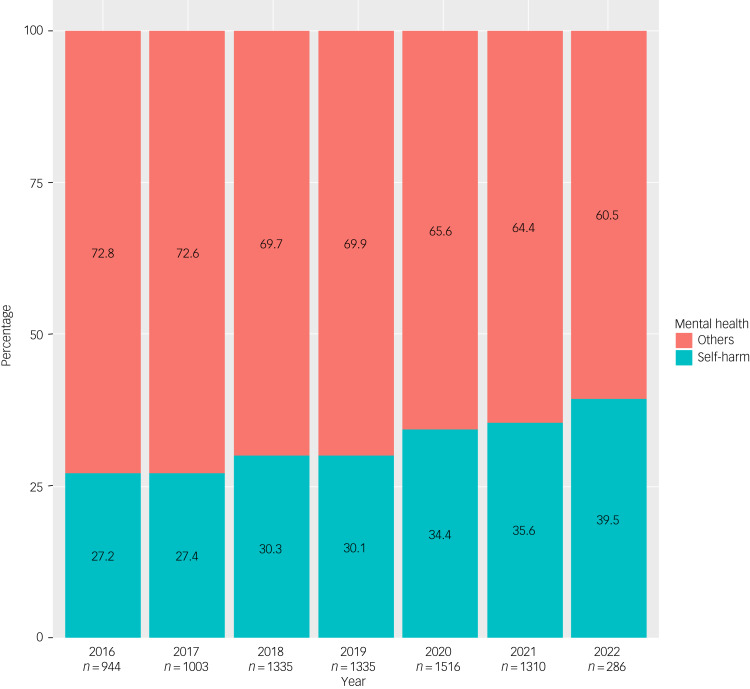
Proportion of self-harm-related mental health emergency department presentations by children and young people (10–17 years of age) between January 2016 and March 2022. The *x*-axis shows the number (*n*) of all emergency department mental health presentations in this age group by year. The data relate to six public hospitals in South Western Sydney, Australia.

The proportion of self-harm-related presentations among all mental health presentations of CYP to emergency department is calculated as row percentages (Table 1). In total, 2457 out of 7789 (31.5%) mental health-associated emergency department presentations were related to self-harm (Table 1). There was also a steady increase in the proportion of self-harm-related mental health presentations to the emergency department from 2016 to 2022 (Fig. 1). The percentage of self-harm-related mental health presentations was higher among female CYP than males (33.7% *v.* 27.4%). However, in terms of CALD status, CYP whose preferred language was not English had about 9% fewer self-harm-related mental health presentations than CYP whose preferred language was English (23.5% *v.* 32%). Similarly, those born in non-English-speaking nations had 4.6% fewer self-harm-related mental health presentations than those born in English-speaking nations (27.7% *v.* 31.8%). The proportion of self-harm-related mental health presentations was higher among CYP residing in socioeconomically disadvantaged areas (quartiles 1–2 = 63% *v.* quartiles 3–4 = 34%). However, CYP from major city areas had a slightly higher proportion of self-harm-related mental health presentations, at 31.5%, compared with those from inner and outer regions (30.5%). The proportion of self-harm-related mental health presentations was higher during weekdays compared to weekends (33.4% *v.* 25.2%). Furthermore, self-harm-related mental health presentations were higher during the COVID-19 period compared to the pre-COVID-19 period (35.7% *v.* 29.1%).

Comparison of clinical features between CALD and non-CALD CYP presenting to the emergency department with self-harm is shown in Table 2. Compared with CYP from a non-CALD background, CYP from a CALD background had a significantly higher proportion of high priority presentations (Triage 1–2) (15.2% *v.* 7.9%) and extended length of stay (75.8% *v.* 68.2%).

**Table 2:** The distribution of clinical features of children and young people of culturally and linguistically diverse (CALD) and non-CALD backgrounds presenting to the emergency department with self-harm only

Clinical features	Non-CALD (*n* = 2258)	CALD (*n* = 198)	*P*
Emergency department to ward			0.427
No	1980 (87.7%)	178 (89.9%)	
Yes	278 (12.3%)	20 (10.1%)	
DAMA			1.000
No	2186 (96.8%)	192 (97.0%)	
Yes	72 (3.2%)	6 (3.0%)	
Intensive care unit			0.217
No	2226 (98.6%)	193 (97.5%)	
Yes	32 (1.4%)	5 (2.5%)	
Triage level			<0.001
High priority (1–2)	179 (7.9%)	30 (15.2%)	
Urgent (3)	1634 (72.4%)	149 (75.3%)	
Low priority (4–5)	445 (19.7%)	19 (9.6%)	
Length of stay, h			0.882
Median	5.729	5.86	
Q1, Q3	3.606, 12.090	4.040, 9.992	
Extended length of stay (≥4 h)			0.031
No	718 (31.8%)	48 (24.2%)	
Yes	1540 (68.2%)	150 (75.8%)	
Waiting time to see by clinician/nurse, h			0.648
Median	0.226	0.237	
Q1, Q3	0.131, 0.397	0.145, 0.428	
Long waiting time to see by clinician/nurse (≥1 h)			1.000
Missing	8	0	
No	2079 (92.4%)	183 (92.4%)	
Yes	171 (7.6%)	15 (7.6%)	
Time of arrival			0.005
00.00–07.59 h	287 (12.7%)	29 (14.6%)	
08.00–17.59 h	1110 (49.2%)	116 (58.6%)	
18.00–23.59 h	861 (38.1%)	53 (26.8%)	
Day of presentation			0.445
Weekday	1838 (81.4%)	166 (83.8%)	
Weekend	420 (18.6%)	32 (16.2%)	

DAMA, Left at own risk (seen by medical staff), Did not wait (not seen by medical staff), or Admitted to emergency department and left at own risk; Q, quartiles.

### Findings of the multilevel logistic regression models

The CALD status associated with self-harm-related mental health emergency department presentation in the regression analysis is presented in Table 3. Based on the unadjusted analysis, it was found that CALD status was associated with lower odds of self-harm-related mental health presentation (COR = 0.82, 95% CI 0.67–0.99). CYP with a non-English preferred language had lower odds of self-harm-related mental health presentation (COR = 0.66, 95% CI 0.50–0.87), even though there was no significant correlation found between country of birth and self-harm-related presentation.

**Table 3:** Relationship between self-harm related mental health emergency department presentation and culturally and linguistically diverse (CALD) status adjusting for covariates and individual-level clustering^a^

Exposure and covariates	Unadjusted models, COR (95% CI)	Adjusted models, AOR (95% CI)			
		Model 1	Model 2	Model 3	Model 4
CALD background					
Yes	0.82* (0.67–0.99)	0.81* (0.66–0.99)			
No	1.00 (reference)	1.00 (reference)			
Country of birth					
Non-English	0.83 (0.66–1.03)		0.82 (0.65–1.02)		0.90 (0.71–1.15)
English	1.00 (reference)		1.00 (reference)		1.00 (reference)
Preferred language					
Non-English	0.66** (0.50–0.87)			0.66** (0.50–0.87)	0.69* (0.51–0.92)
English	1.00 (reference)			1.00 (reference)	1.00 (reference)
Age at emergency department presentation, years	1.04* (1.01–1.07)	1.05** (1.02–1.08)	1.05** (1.02–1.09)	1.05** (1.02–1.08)	1.05** (1.02–1.08)
Gender					
Female	1.41*** (1.25–1.58)	1.38*** (1.22–1.55)	1.38*** (1.22–1.55)	1.37*** (1.21–1.54)	1.37*** (1.22–1.54)
Male	1.00 (reference)	1.00 (reference)	1.00 (reference)	1.00 (reference)	1.00 (reference)
Day of arrival					
Weekend	0.65*** (0.57–0.74)	0.65*** (0.57–0.75)	0.66*** (0.58–0.75)	0.65*** (0.57–0.74)	0.65*** (0.57–0.75)
Weekday	1.00 (reference)	1.00 (reference)	1.00 (reference)	1.00 (reference)	1.00 (reference)
Residential area socioeconomic status					
Q1: Least advantaged (1–25)	1.01 (0.87–1.17)	1.03 (0.88–1.20)	1.02 (0.88–1.19)	1.03 (0.89–1.20)	1.04 (0.89–1.21)
Q2: 2nd least advantaged (26–50)	1.07 (0.88–1.31)	1.13 (0.92–1.38)	1.13 (0.92–1.38)	1.12 (0.91–1.36)	1.12 (0.92–1.37)
Q3: 2nd most advantaged (51–75)	0.97 (0.80–1.17)	1.01 (0.83–1.23)	1.01 (0.83–1.24)	1.01 (0.83–1.23)	1.01 (0.83–1.24)
Q4: Most advantaged (76–100)	1.00 (reference)	1.00 (reference)	1.00 (reference)	1.00 (reference)	1.00 (reference)
Area remoteness					
Inner or outer region	0.96 (0.81–1.13)	0.95 (0.78–1.14)	0.95 (0.78–1.15)	0.95 (0.78–1.15)	0.94 (0.78–1.14)
Major city	1.00 (reference)	1.00 (reference)	1.00 (reference)	1.00 (reference)	1.00 (reference)
COVID period					
During COVID-19	1.39*** (1.24–1.55)	1.38*** (1.23–1.54)	1.38*** (1.23–1.54)	1.37*** (1.23–1.54)	1.38*** (1.23–1.54)
Pre-COVID-19	1.00 (reference)	1.00 (reference)	1.00 (reference)	1.00 (reference)	1.00 (reference)
Patients, *n*		7550	7539	7540	7529

a. Unadjusted and adjusted models were considered patient-level clustering.

COR, crude odds ratio based on unadjusted models; AOR, adjusted odds ratio based on adjusted models; Q, quartiles.

*P < 0.05, **P < 0.01, ***P < 0.001.

Consistent with the results of unadjusted models, CYP from a CALD background had 19% lower odds (AOR = 0.81, 95% CI 0.66–0.99) of having a self-harm-related mental health presentation in adjusted models. Preferred language was found to be associated with lower odds of self-harm-related mental health presentations in the adjusted model (AOR = 0.66, 95% CI 0.50–0.87). In contrast, country of birth did not demonstrate a significant association with self-harm-related mental health presentations, and these findings are consistent with the results obtained from unadjusted models as well. When both preferred language and country of birth were incorporated into an adjusted model, no notable variations in the magnitudes of associations were observed. It is noteworthy that the value of the variance inflation factor (VIF) statistics did not reveal any significant multicollinearity (results are not shown).

In both unadjusted and adjusted analyses, the following covariates demonstrated a significant correlation with self-harm-related mental health emergency department presentation: age at presentation, gender, day of arrival and COVID-19 period. In both the models, older adolescents and females had higher odds of having self-harm-related mental health presentations than younger and male CYP respectively. The odds of self-harm presentation were significantly lower on weekends compared with weekdays. Compared with pre-COVID-19, the odds of self-harm-related mental health emergency department presentation increased during the COVID-19 period.

## Discussion

### Summary of the findings

In this study, we undertook an analysis of self-harm-related mental health presentations to emergency departments by children and young people (CYP) aged 10–17 years, in six public hospitals in the South Western Sydney Local Health District (SWSLHD) from 2016 to 2022. We found a steady increase in the proportion of self-harm-related mental health presentations to emergency departments over the 6-year study period, from 27 to 39%. CALD background was associated with lower odds of presenting to the mental health emergency department for self-harm, after adjusting for potential covariates and individual-level clustering. There were also significant associations between self-harm-related mental health emergency department presentations and older age, female gender, weekday presentations and the COVID-19 period.

### Comparison with existing literature

Almost one in three (31.5%) of all mental health-associated emergency department presentations by CYP were related to self-harm, alongside a steady increase during the study period. The increasing number of self-harm presentations to the emergency department by CYP observed in this study is consistent with the recent literature.^16,24^ This worsened trend might be attributed to the effects of the COVID-19 pandemic, as our study, consistent with local^16,24^ and international evidence,^25^ found that the pandemic period was significantly associated with higher odds of presentation. These exacerbated rates of self-harm presentation among CYP might be due to school closures, lack of social gatherings, family stress and domestic violence.^6^

Findings of this study particularly looked at the association of self-harm-related mental health emergency department presentations among CYP from culturally and linguistically diverse (CALD) backgrounds. We found that CYP from a CALD background – either CALD status (country of birth and/or preferred language) or preferred language only (non-English) – had lower odds of presenting to the emergency department for self-harm compared with those from a non-CALD English-language background. However, CALD status based on country of birth was not a significant factor. This may be due to the fact that many of the children born in Australia have families from CALD backgrounds. Evidence on CALD status and self-harm remains unclear, with some studies reporting higher rates of presentation, particularly among refugees and asylum seekers.^13,26^ However, other studies have shown reduced presentations among CALD groups owing stigma around mental illness within families and the community,^27^ concerns about care provision and language barriers.^28^ Additionally, CALD CYP's experiences of discrimination or racism and their struggle with a sense of belonging can contribute to risk of self-harm and health service avoidance.^29^ Further, other studies report that CALD CYP's social networks and supports, religious beliefs and individual resilience could be some of the protective factors against self-harm.^30,31^ Nevertheless, further investigation is needed to determine whether the low rates of presentation from CALD backgrounds reflect actual lower rates, or are due to stigma and/or differences in help-seeking behaviours. Additionally, research to better understand the variation by taking into account key factors such as the mode of entry into Australia (refugee versus planned migration), complemented by detailed qualitative interviews of CALD CYP, is critical. This will deepen the understanding about how different self-harm prevention strategies are needed for different CALD populations and aid in better supporting the specific needs of CYP from CALD communities most affected by self-harm.

Consistent with the findings of other studies,^24,32^ we found key sociodemographic factors such as older age and female gender to be significant predictors of self-harm-related emergency department presentations. The predominance of female adolescents with increased presentations has been well established, with a previous study reporting 6:1 and 3.5:1 female to male ratios at 12–14 years and 15–17 years respectively.^33^ This is also reflected in our descriptive findings, where the numbers of female adolescents presenting for overall mental health problems and self-harm were double those of males. Although it could be true that female adolescents could have higher mental health service use than male adolescents, this could also be due to higher rates of help-seeking behaviours and readiness to discuss emotional problems among female adolescents compared with males.^34^ However, other risk factors for this gender inequality may also include higher reports of poor self-image, bullying at school and via social media, academic pressure, abuse, and neglect at home.^35^ Hence, it is essential to continue considering gender-related differences and characteristics to better understand self-harm behaviours in the adolescent population.

Interestingly, we also found that the weekdays (compared with weekends) were significantly associated with higher odds of self-harm-related mental health emergency department presentations among CYP. Only a few studies have reported on the timing of self-harm-related emergency department presentations among CYP, of which Sara et al's study^16^ mentioned that presentations peaked on Mondays and declined during the week to a low on Saturdays. In line with this, Colman et al's study^36^ also reported that Sundays and Mondays were the most common days for mental health presentations. This may partially be attributable to fewer out-patient services being available during the weekend. It may be speculated that this may be also due to CYP being more likely to encounter an adverse event relating to friendship or relationship issues, as well as academic stresses, which might trigger self-harm behaviours while at school on weekdays, compared with the weekends when they are with families. Furthermore, on weekends, CYP may spend more time with their families, which can serve as a protective factor against self-harm-related behaviours by providing family support.^37^

### Methodological reflections

Our study has several strengths. The data are drawn from a large clinical and administrative data-set from six public hospitals in SWSLHD, an area of significant social and economic disadvantage and high proportion of CALD population groups. This study used an encounter-level analysis as opposed to a patient-level analysis, making it possible to evaluate encounter-level factors in relation to self-harm-related presentations. The findings of this study emphasise the importance of enhancing culturally appropriate mental health services in the community, to alleviate the burden associated with self-harm among adolescents in SWSLHD. We also note several limitations to our study. First, our study is based on data from six public hospitals, hence we cannot comment on encounters in the community or emergency department presentations to other hospitals outside the local health district. Second, the findings demonstrate an association rather than a causative relationship between predictors and outcomes. Third, we defined the CALD status using the Australian Bureau of Statistics’ minimum core set of cultural and language indicators, such as preferred language and/or country of birth. However, we acknowledge that there are other identifiers for CALD status, for instance the reason for migration (e.g. skilled migration versus refugee/asylum seekers). Hence, the decreased odds of self-harm presentation among the CALD communities may not be a true representation of different social and ethnic groups. Further, the association between CALD status and self-harm might be attributable to unmeasured confounders, and hence their relationship needs to be interpreted with caution. Finally, unlike a study^38^ which explored the type of self-harm (suicidal attempts versus non-suicidal self-injuries), this information along information on repeat presentations was not available for analysis, given the nature of the administrative data. Hence, there is opportunity for future research to further explore in detail how people of different CALD backgrounds seek health services for different mental health issues and the mental health service needs they have.

### Implications of the findings

Our study found a year-on-year increase in the proportion of self-harm-related emergency department presentations by CYP in SWSLHD. Although our primary analysis showed lower odds of self-harm-related emergency department presentations by CYP from a CALD background, other risk factors, such as older age, female gender and the pandemic period, were associated with higher odds of presentation. Nevertheless, these findings demonstrate that if service provision has remained static over this time, then the increasing demand may be challenging the capacity of services.^22^ Further, Geulayov et al^39^ reported on the iceberg model of self-harm, where among CYP in England the rate of community-reported self-harm was 10 times higher than the rate of hospital-presenting self-harm. Thus, the true extent of the overall prevalence of self-harm is likely to be much higher in the community than that reflected in the emergency department presentations reported here. In line with this, the recent establishment in Australia of the National Mental Health Commission's National Suicide and Self-Harm Monitoring System may assist in defining/measuring self-harm presentations in the future.^40^ With individuals and families finding it increasingly difficult to access and navigate suitable services (hence ending up in the emergency department for acute care), there is an urgent need to implement innovative service models that are responsive, integrated, equitable and sustainable.

Alternative service models to the traditional emergency department and hospital-based service delivery also deserve attention in this regard. In line with this, NSW Health's recent implementation of alternative models of care for emergency department avoidance, such as Towards Zero Suicide (TZS) and the ‘Safeguards’ child and adolescent mental health rapid response teams, may help redirect the patient load away from emergency departments. Further, targeted supports in a less formal non-emergency department community setting, including the involvement of people with lived experience as peer workers, may be preferred by CYP and such models would benefit from robust evaluation.

## Supporting information

John et al. supplementary material John et al. supplementary material

## Data Availability

Data are available on formal request to the BestSTART Data Observatory Research Governance Team (https://www.beststartsws.org.au/).
