# Supplementary material for: Self-harm-related mental health presentations to emergency departments by children and young people from culturally and linguistically diverse groups in South Western Sydney
Source: BJPsych Open. 2024 Dec 4;10(6):e213. doi: 10.1192/bjo.2024.763 (PMC11698175; doi:10.1192/bjo.2024.763)
Supplement: John et al. supplementary material [file S2056472424007634sup001.docx]

**Supplementary table 1. ICD-10 AM codes and their descriptors used in this study**

| **ICD-10 AM code** | **Descriptor** |
| --- | --- |
| X60 | Intentional self-poisoning by and exposure to nonopioid analgesics, antipyretics and antirheumatics |
| X61 | Intentional self-poisoning by and exposure to antiepileptic, sedative-hypnotic, ant parkinsonism and psychotropic drugs, not elsewhere classified |
| X62 | Intentional self-poisoning by and exposure to narcotics and psychodysleptics [hallucinogens], not elsewhere classified |
| X63 | Intentional self-poisoning by and exposure to other drugs acting on the autonomic nervous system |
| X64 | Intentional self-poisoning by and exposure to other and unspecified drugs, medicaments and biological substances |
| X65 | Intentional self-poisoning by and exposure to alcohol |
| X66 | Intentional self-poisoning by and exposure to organic solvents and halogenated hydrocarbons and their vapours |
| X671 | Intentional self-poisoning by and exposure to carbon monoxide from utility gas |
| X672 | Intentional self-poisoning by and exposure to carbon monoxide from other domestic fuels |
| X678 | Intentional self-poisoning by and exposure to other specified gases or vapours |
| X679 | Intentional self-poisoning by and exposure to unspecified gases or vapours |
| X68 | Intentional self-poisoning by and exposure to pesticides |
| X69 | Intentional self-poisoning by and exposure to other and unspecified chemicals and noxious substances |
| X700 | Intentional self-harm by hanging |
| X701 | Intentional self-harm by strangulation |
| X702 | Intentional self-harm by suffocation |
| X710 | Intentional self-harm by drowning and submersion in bath-tub |
| X718 | Intentional self-harm by drowning and submersion in other specified water |
| X719 | Intentional self-harm by drowning and submersion in unspecified water |
| X76 | Intentional self-harm by smoke, fire and flames |
| X77 | Intentional self-harm by steam, hot vapours and hot objects |
| X780 | Intentional self-harm by knife |
| X781 | Intentional self-harm by razor blade |
| X782 | Intentional self-harm by hypodermic needle and syringe |
| X783 | Intentional self-harm by glass |
| X788 | Intentional self-harm by other specified sharp object |
| X789 | Intentional self-harm by sharp object, unspecified |
| X79 | Intentional self-harm by blunt object |
| X80 | Intentional self-harm by jumping from a high place |
| X810 | Intentional self-harm by jumping or lying before a train |
| X811 | Intentional self-harm by jumping or lying before a tram |
| X812 | Intentional self-harm by jumping or lying before a motor vehicle |
| X818 | Intentional self-harm by jumping or lying before other specified moving object |
| X819 | Intentional self-harm by jumping or lying before unspecified moving object |
| X83 | Intentional self-harm by other specified means |
| X84 | Intentional self-harm by unspecified means |
| Y10 | Poisoning by and exposure to nonopioid analgesics, antipyretics and antirheumatics, undetermined intent |
| Y11 | Poisoning by and exposure to antiepileptic, sedative-hypnotic, antiparkinsonism and psychotropic drugs, not elsewhere classified, undetermined intent |
| Y12 | Poisoning by and exposure to narcotics and psychodysleptics [hallucinogens], not elsewhere classified, undetermined intent |
| Y13 | Poisoning by and exposure to other drugs acting on the autonomic nervous system, undetermined intent |
| Y14 | Poisoning by and exposure to other and unspecified drugs, medicaments and biological substances, undetermined intent |
| Y15 | Poisoning by and exposure to alcohol, undetermined intent |
| Y16 | Poisoning by and exposure to organic solvents and halogenated hydrocarbons and their vapours, undetermined intent |
| Y170 | Poisoning by and exposure to carbon monoxide from combustion engine exhaust, undetermined intent |
| Y171 | Poisoning by and exposure to carbon monoxide from utility gas, undetermined intent |
| Y172 | Poisoning by and exposure to carbon monoxide from other domestic fuels, undetermined intent |
| Y178 | Poisoning by and exposure to other specified gases or vapours, undetermined intent |
| Y179 | Poisoning by and exposure to unspecified gases or vapours, undetermined intent |
| Y18 | Poisoning by and exposure to pesticides, undetermined intent |
| Y19 | Poisoning by and exposure to other and unspecified chemicals and noxious substances, undetermined intent |
| Y20 | Hanging, strangulation and suffocation, undetermined intent |
| Y210 | Drowning and submersion in bath-tub, undetermined intent |
| Y218 | Drowning and submersion in other specified water, undetermined intent |
| Y219 | Drowning and submersion in unspecified water, undetermined intent |
| Y22 | Handgun discharge, undetermined intent |
| Y241 | Air rifle discharge, undetermined intent |
| Y242 | Shotgun discharge, undetermined intent |
| Y243 | Small calibre rifle discharge, undetermined intent |
| Y244 | Large calibre rifle discharge, undetermined intent |
| Y249 | Discharge from other and unspecified firearms, undetermined intent |
| Y25 | Contact with explosive material, undetermined intent |
| Y26 | Exposure to smoke, fire and flames, undetermined intent |
| Y27 | Contact with steam, hot vapours and hot objects, undetermined intent |
| Y280 | Contact with knife, undetermined intent |
| Y281 | Contact with razor blade, undetermined intent |
| Y282 | Contact with hypodermic needle and syringe, undetermined intent |
| Y283 | Contact with glass, undetermined intent |
| Y288 | Contact with other specified sharp object, undetermined intent |
| Y289 | Contact with unspecified sharp object, undetermined intent |
| Y29 | Contact with blunt object, undetermined intent |
| Y30 | Falling, jumping or pushed from a high place, undetermined intent |
| Y310 | Falling, lying or running before or into a train, undetermined intent |
| Y311 | Falling, lying or running before or into a tram, undetermined intent |
| Y312 | Fall lie move motor vehicle undet intent |
| Y318 | Falling, lying or running before or into other specified moving object, undetermined intent |
| Y319 | Falling, lying or running before or into unspecified moving object, undetermined intent |
| Y33 | Other specified events, undetermined intent |
| Y34 | Unspecified event, undetermined intent |
| Y87 | Sequelae of intentional self-harm |
| Y87.2 | Sequelae of events of undetermined intent |
| Z91.5 | Personal history of self-harm (Parasuicide, Self-poisoning, and Suicide attempt) |
| R45.81 | Suicidal ideation (except with underlying mental or behavioural disorder) |
